# Can an organizationally anchored, multilevel intervention improve perceived stress and psychosocial factors in the workplace? A pre-post study assessing effectiveness and implementation

**DOI:** 10.1186/s12889-024-20801-5

**Published:** 2025-01-30

**Authors:** Vita Ligaya Dalgaard, Tanja Kirkegaard, Christian Dyrlund Wåhlin-Jacobsen, Birgit Aust, Sofie Jaspers, Thomas Faurholt Jønsson, Trine Nøhr Winding

**Affiliations:** 1 Department of Psychology and Behavioral Sciences, Aarhus University, Aarhus, Denmark; 2 Department of Organization, Copenhagen Business School, Copenhagen, Denmark; 3 The National Research Centre for the Working Environment, Copenhagen, Denmark; 4 Department of Occupational Medicine, University Research Clinic, Danish Ramazzini Centre, Goedstrup Hospital, Herning, Denmark; 5 Department of Communication and Psychology, Aalborg University, Aalborg, Denmark

**Keywords:** Perceived stress, Psychosocial work environment, Organizational, Multilevel intervention, Organizational intervention, Job satisfaction, Psychosocial safety climate, Participatory

## Abstract

**Background:**

Organizational multilevel interventions have been called for as a means to improve psychosocial working conditions, reduce stress, and enhance wellbeing in organizations. However, these types of interventions are highly complex to implement and evaluate, and they remain scarce in the literature. In this study, we present the evaluation of a multilevel intervention conducted in a municipality setting.

**Methods:**

The intervention was based on a train-the-trainer principle and participatory risk assessment workshops on all organizational levels. Action plans were subsequently developed at the team level, and identified risks, which could not be addressed at the team level, were reported to the management for further action planning or escalation to the next management level. Using a pre-post study design, we evaluated changes in proximal outcomes related to the psychosocial working environment, and distal outcomes related to stress and job satisfaction. Changes over time in outcome measures were analyzed using linear mixed models. A quantitative process evaluation was used to examine the degree of implementation of the intervention.

**Results:**

Small improvements over time were observed with regard to quantitative demands, overall perception of the psychosocial work environment, job satisfaction, and the psychosocial safety climate. We also observed an increase in empowering leadership. Positive tendencies were also found for predicatability at work, possibilities for solving work tasks and support from closest manager. The effect sizes were small in all cases. No improvements in perceived stress or stress symptoms were found. The study revealed several practical and methodological challenges in conducting and implementing this type of multilevel intervention in a municipal setting.

**Conclusions:**

Overall, our study suggests that the intervention was associated with small positive changes in certain aspects of the working environment but no improvements were observed in stress outcomes. The study highlights a number of challenges in relation to implementing this type of multilevel intervention in a municipal setting.

**Trial Registration:**

The study was prospectively registered at ISRCTN84940247 on April 23, 2019.

**Supplementary Information:**

The online version contains supplementary material available at 10.1186/s12889-024-20801-5.

## Background

### Work-related stress

Work-related stress is widespread in Western countries [1] and one of the most important public health concerns facing modern workplaces [2]. From a psychological viewpoint, stress can be understood as the experience of an imbalance between perceived demands of a situation and available resources [3]. Work-related stress pertains to situations where this experience is related to the work context (i.e. high workload or other factors at work) [4]. Ongoing stress may over time develop into mental health problems such as adjustment disorder or depression [5, 6]. Such mental health conditions have been associated with sickness absence [5, 7], reduced income [8], and negative health consequences such as cognitive difficulties [9, 10], sleep disturbance, [11],and cardiovascular disease [12]. The human suffering related to stress is high for employees, their employers and society [13]. Finding effective solutions to reduce work-related stress is therefore crucial.

### The psychosocial work environment

The psychosocial work environment is an important determinant of mental health outcomes [14] and consists of elements such as organizational climate, social relations at work, and the design and organization of work [15]. One theoretical approach to understanding the relationship between the psychosocial work environment and work-related stress/burnout is the Job Demands-Resources model (JD-R), which builds on previous models like the Job-Demand Control model [16] and the effort-reward imbalance model [17]. The JD-R model emphasizes that a suitable balance between resources (e.g., supportive relationships at work) and demands (e.g., the number of work assignments or difficult interactions) helps prevent stress and burnout and enhance employee engagement. Recent updates to the JD-R also contribute with a multilevel perspective, where the employee is seen as nested in teams imbedded within organizations, and where top management and HR practices shape the organizational climate [18, 19]. Therefore, preventive interventions may benefit not only from addressing the balance between job demands and resources for each employee, but also from targeting organizational management levels as a means to reduce stress and improve employee well-being.

### Organizational level Interventions for improving employee health and well-being

In line with the broader organizational focus of the JD-R, researchers have argued that interventions for improving employee health and well-being must move beyond targeting symptomatic individuals alone. Although such individual level interventions have proven effective [20], researchers have emphasized that interventions aimed at reducing employee stress and enhancing well-being should prioritize fostering a healthy psychosocial working environment [21–23]. As such, it has been recommended that efforts to prevent work-related mental health problems should target the organizational level [24, 25]. In particular, a multilevel approach including organizational levels such as the work group, management and organizational support functions has been called for since many sources of strain may originate at these levels. For example, Martin et al. [22] advocated that all organizational levels should be taken into account, and Nielsen and Christensen [26] argued that participatory organizational interventions should be developed on the basis of the entire IGLO framework involving the individual, group, leader and organizational levels.

Organizational interventions aim to address working conditions and how work tasks are planned, and structured. These interventions typically follow a stepwise approach containing phases of preparation, identification of problem areas followed by the development, implementation and evaluation of action plans [27]. Recently, an extensive review of reviews identified 52 reviews covering 957 studies and concluded that the work environment and health outcomes of employees can be improved through certain organizational interventions (e.g., changes related to working time) [28]. In addition, organizational interventions are generally believed to address more sustainable changes in working conditions and employee well-being [21, 29]. By leveraging internal resources, organizations reduce reliance on external researchers and consultants, ensuring that knowledge and expertise remain within the organization, rather than being lost when external parties depart after the project period [25]. Furthermore, these interventions often activate line managers to implement new initiatives across the organization. Line managers are known to play a critical role in the implementation of organizational interventions [30, 31] and in promoting a healthy work environment in general [32, 33].

Another typical component of organizational interventions is employee involvement. Since employees know how daily work is performed, they are able to assess whether considered initiatives are viable, and they can help design solutions that appropriately fit the organization [25, 27]. Employee involvement may also contribute to a shared understanding of work environment issues, which is a prerequisite for successful action planning and implementation [34]. Finally, the involvement of employees in the development and implementation of interventions may directly affect employee health and well-being through increases in job control [35] or perceived justice within the organization [36]. Relatedly, a high degree of involvement is considered a basic principle within the psychosocial safety climate model (PSC), which outlines how organizations prioritize the protection of psychological health and safety through policies, practices and procedures and involvement of all stakeholders and parts of the organization. Research has found that PSC can predict both the positive development of the local work environment and the successful implementation of new initiatives [18, 37]. The current study is built upon an organizationally anchored participatory and multilevel approach that integrates many of these key findings from the existing research and hence follows research based recommendations.

### Challenges for organizational level interventions

There are also a number of important caveats in relation to organizational interventions. The effects of organizational interventions have been found to be inconsistent, potentially because of circumstances such as unsuccessful implementation or inadequate fit with the specific context in which the intervention is tested [20, 38–41]. Organizational interventions are complex and demanding of the organizations in which they take place [31, 42], and a wide range of factors are known to affect the implementation process [43]. As a result, they do not always go as planned [44], and may in some cases even lead to negative effects on employee health and well-being [31, 45]. Some of these challenges are specifically linked to employee involvement; for example, as noted by Aust et al. [31], employees’ expectations towards the changes that can be achieved through the intervention may be disappointed. Others have mentioned that interventions may end up focusing solely on local issues, rather than more fundamental problems that affect employees throughout the organization [46]. Employees may also feel that they are made responsible for existing work environment problems without the requisite resources to address these problems [47].

Other challenges relate more closely to the methodological aspects of conducting multilevel intervention studies. As contexts for intervention, workplaces are constantly changing and, in contrast to typical experimental settings, not controlled by the researcher. Therefore, the chosen outcome measures may be affected by various simultaneous processes unrelated to the intervention, violating central assumptions connected with randomized and/or controlled designs [47, 48]. Even in cases where randomization of units is possible, it often takes place at a high organizational level (e.g., entire organizations or departments), which limits the number of units that can be randomized. Few units reduce the likelihood that confounders will be equally distributed among the intervention and control groups and that the effects of unforeseen contextual events will even out across groups. In addition, the contextual specificity of the observed effects means that results cannot necessarily be generalized to other organizations, even when seemingly similar [25, 49]. Interventions are often fitted specifically to the organizational context, and even when following the same intervention principles, different mechanisms may be triggered across settings [50]. Because of these challenges, organizational intervention studies often focus on the effectiveness of the intervention under the given circumstances rather than the more general efficacy of the intervention method [51]. Thus, the collection of process evaluation data is needed for a nuanced assessment of what can be inferred from the study [52]. Process evaluation data may be used in combination with effect evaluation data to assess if contextual or processual factors may have played a role in the overall implementation and effectiveness of the intervention [53–55].

### Aims

The aims of this study are to 1) evaluate pre-post changes in perceived stress and relevant psychosocial working environment indicators following an organizationally anchored multilevel intervention, and 2) based on descriptive statistics, to examine the degree to which the intervention was implemented and whether implementation factors (such as participation in workshops, degree of implantation of action plans) could have played a role in the results.

The intervention is grounded in several principles recommended in the literature, including employee participation through a number of workshops and subsequent work with action plans, utilization of existing organizational resources, and implementation at multiple organizational levels. The intervention features an innovative approach that has not previously been evaluated according to scientific standards. A detailed description of the intervention is presented in the methods section.

## Materials & Methods

### Study design and participants

#### Workplace recruitment and withdrawal

This evaluation study intended to evaluate an organizationally anchored multilevel intervention in both private and public organizations. Twenty-three public and private organizations were contacted as part of the early recruitment process before obtaining agreements to participate with two organizations. The two organizations were a public municipality and a private consumer electronics company. In both organizations, the decision to participate was made at the administrative level after consulting top management. However, early in the intervention process, the private company decided to withdraw from the study due to major changes in top management. Because the withdrawal happened shortly after the first workshops had been conducted and before any action plans were developed, we decided to not include the private company in this evaluation study and to focus solely on the intervention conducted in the municipality.

#### Recruitment of workplaces within the participating municipality

The present intervention study reports on the intervention as it was implemented in a Danish municipality under the section of Children and Youth, one of four municipality sections. Participants comprised employees and managers from daycare institutions, schools and administrative units.

As part of recruiting workplaces and planning the intervention within the municipality, initial meetings between the researchers and the Human Resource Department (HR) took place followed by a meeting where top-level managers also took part, and where the goal, structure and activities of the intervention were discussed. Based on these meetings, it was decided that workplaces/departments should be recruited among schools, daycare centers and family departments. The municipality's HR department was responsible for recruitment and contacted all relevant institutions within the children and youth section by mail and invited them to participate in an intervention study with the focus of improving the psychosocial work environment and reducing stress. The invitation letter also contained an invitation for department managers to participate in an information meeting to learn about the project. During this recruitment process, two other departments (visitation and counseling) from another area of the municipality became interested in the project and were allowed to participate. Participation of each workplace in the study was decided by the individual managers of the workplace. A detailed presentation of participant characteristics is provided in Table 1.

**Table 1:**
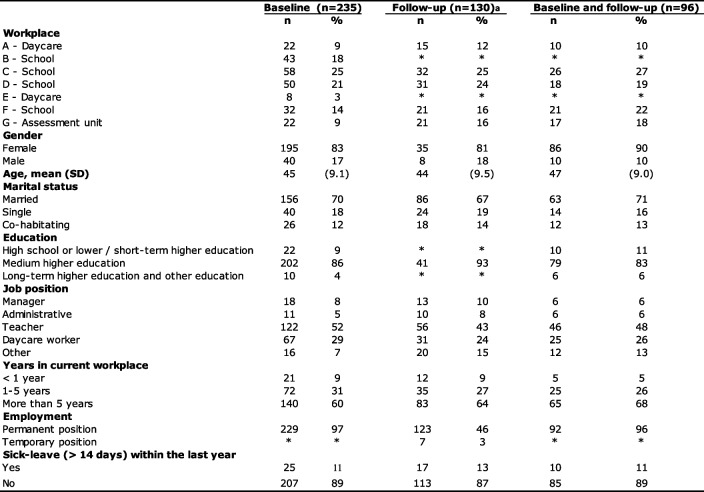
Characteristics and demographics of all who participated at baseline (*n* = 235) and those who participated at follow-up (*n* = 130) and those who participated at both baseline and follow-up (*n* = 96)

Missing values are not depicted in the table due to small numbers

*Represents cells with 5 observations or less or cells with information that reveal small numbers in other cells

^a^Numbers are small with regard to gender and education because only those who had not previously provided this information were asked at the follow-up

### The intervention

#### Origin of the intervention and adaptation to the municipal context

An early version of the intervention evaluated in this study was developed by HR-personnel at a large international pharmaceutical company to target and prevent stress in response to an employee survey that revealed high levels of stress symptoms [56]. Although not scientifically evaluated, the HR team presented their evaluation at a Danish research forum. The intervention consisted of participatory risk assessment and action planning at the team level, with unresolved issues escalated to higher management. A novel feature was the involvement of all management levels, allowing for the escalation of issues to the next managerial level, when local managers lacked the authority to resolve them. This IGLO-based approach (Individual, Group, Leader, Organization) including existing human resource management systems and organizational policies (i.e., IGLO levels; see Nielsen et al. [57]) inspired the authors of the current paper to adapt and test the intervention in other settings for research purposes. Initially the research group met with the HR personnel from the pharmaceutical company and they provided information on their experience with the intervention and from this point of departure, the version of the intervention tested in this study was developed by the researchers.

To adapt the intervention to the municipal context, the researchers developed introductory materials for the HR personnel of the municipality to equip them with skills to train the managers in the municipality. A presentation of the psychosocial work environment, which was part of the original material was turned into a video lecture by the researchers. In the original material, managers could choose from six dialogue-based tools to engage with employees, but we selected the Delphi dialogue tool [58] as the only tool they could use to simplify the process for participants. Additionally, we simplified the IGLO action plan to make it more user-friendly and developed accessible material on its content, including speaker notes for slides, to facilitate participants understanding and application of the method. The adapted intervention is described as it was planned below.

#### Content of the municipal intervention

The planned intervention, as adapted to the municipality, includes training seminars and participatory workshops where research-based insights on the psychosocial work environment and stress are presented to develop a shared understanding. A video (as mentioned above) produced by the research team on key work-related stressors, is shown at each session, with managers presenting it during their workshops with employees. Each seminar and workshop incorporates two core tools: A Delphi dialogue tool [58], used to identify both positive and problematic aspects of the work environment, and an IGLO-based action template [26]. These tools are introduced during the training seminars and employed in the participatory workshops to guide discussions and planning.

The planned invention includes the following principles:

- *Train the Trainer*: HR personnel are trained by researchers to train managers in facilitating team dialogues with a point of departure in a dialogue-based mapping tool (Delphi tool) as well as IGLO based actions plans.
- *Team dialogues*: All managers use a Delphi dialogue tool and IGLO action plans in their team and among managers themselves.
- *Escalation*: Action plans or problems that cannot be resolved at the existing manager level are escalated to the next management level by managers.
- *Collection*: All IGLO action plans are sent to an HR mailbox after the team sessions. HR personnel place themes and issues at the relevant organizational level and identify general trends to be presented to top management. This is a parallel process and these results are communicated initially to top management and subsequently to all personnel at the end of the escalation process.
- *Consolidation*: All organizational levels work with IGLO plans

**Fig. 1:**
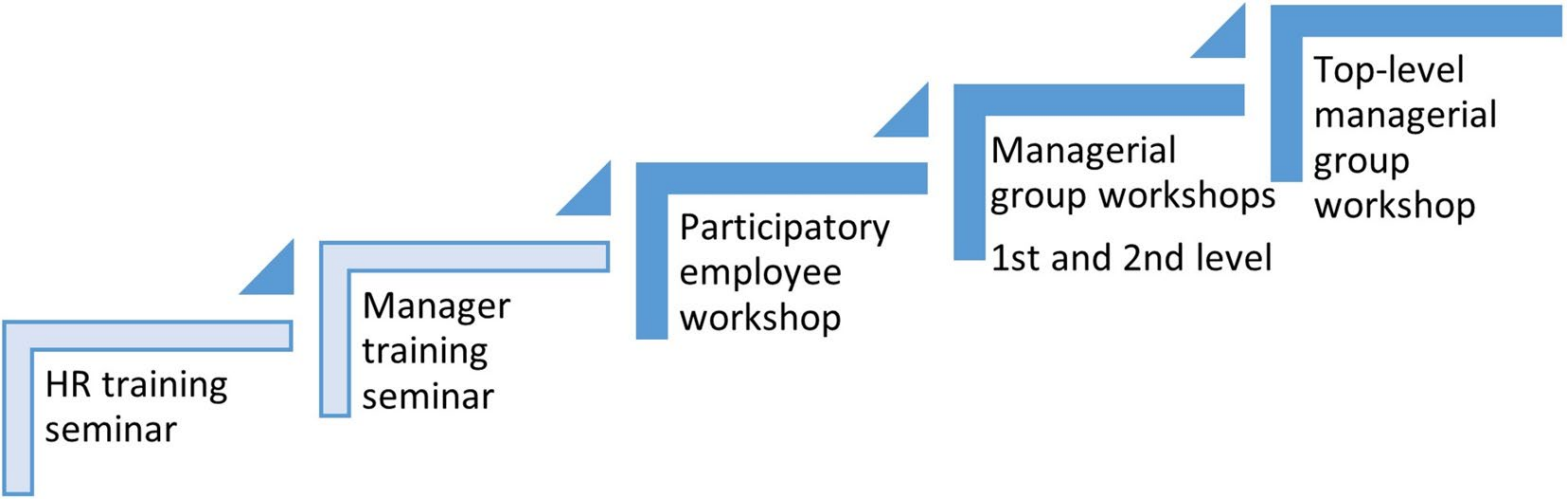
Illustration of the train the trainer principle and levels targeted by the intervention

An overview of the planned intervention steps are shown in Fig. 1 and explained in detail below.

#### Elaboration of intervention activities

During the early stages, a steering committee of HR personnel from the municipality and research group members is established. A meeting between HR, the research group and workplace managers follow to introduce the project purpose.

Intervention activities are planned to take place at different time points with a progression of participation at multiple organizational layers, as illustrated in Fig. 1 above.

The train the trainer principle [59] is employed during the first part of the intervention which consists of *a 3-hour training seminar only for HR personnel.* Here, the research team presents all the materials for the manager training seminar and for the employee and manager workshops. The seminar prepares HR to train managers in facilitating and communicating with employees using the Delphi tool and IGLO action plans. In addition, HR is instructed to collect and categorize action plans from multiple worksites and at different organizational levels throughout the project period. The research team provides a simple database to assist HR members in structuring the chosen topics and to help keep track of the implementation of chosen action plans.

**Fig. 2:**
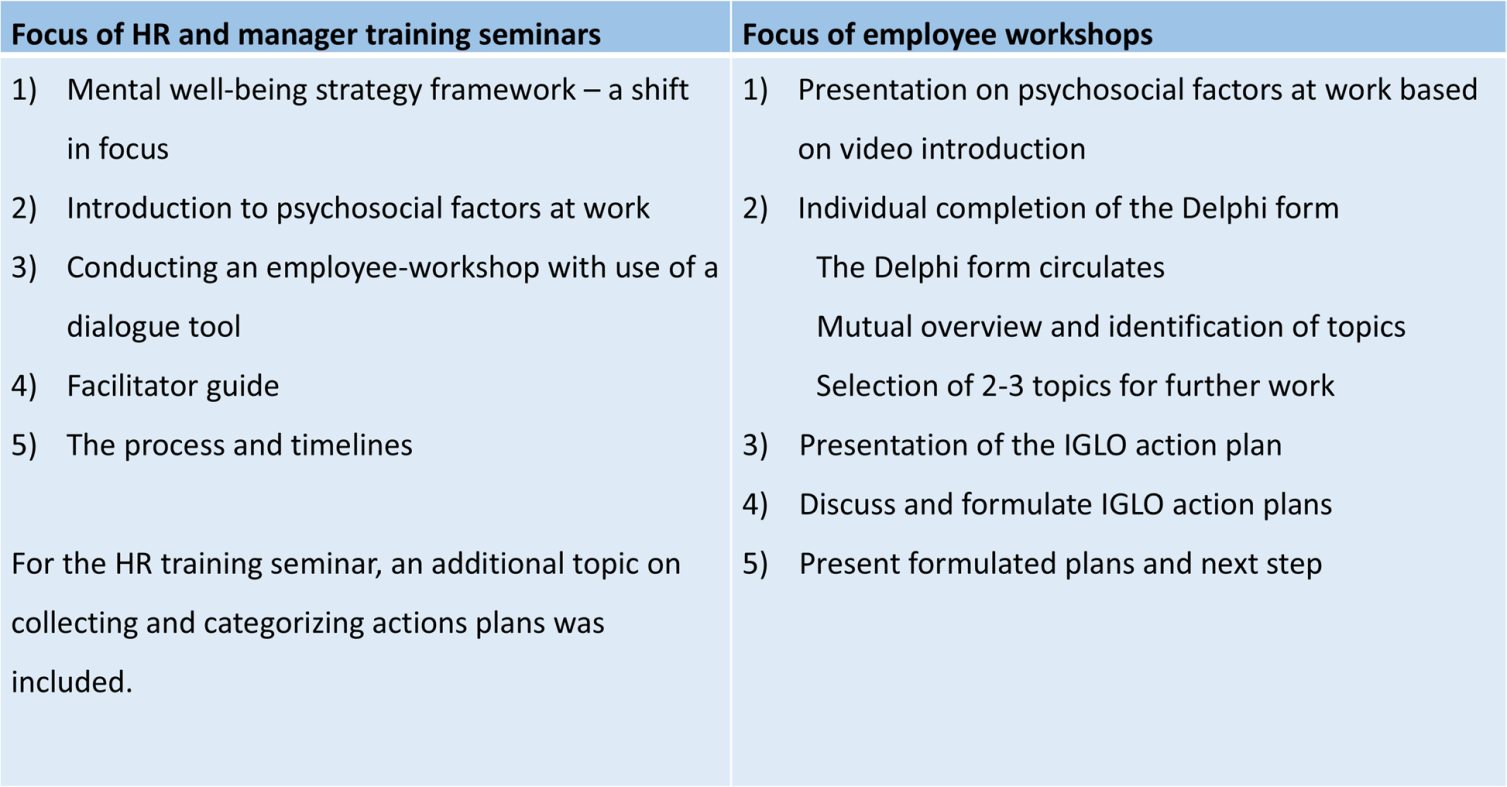
Overview of the agenda at the intervention seminars and workshops

This is followed by the *2.5-hour manager training seminar* (see Fig. 1 above and Fig. 2 below), where HR presents the material to the managers who are given individual folders with all material, including a list of their roles and responsibilities during the intervention phase.

Subsequently, managers are asked to conduct *a 2.5-hour participatory workshop* with their employees, where the managers present the project material. Moreover, the managers use the Delphi dialogue tool (see a detailed description below) to facilitate the process and prioritize topics into IGLO-action plans. Managers were responsible for taking copies of all employee action plans, sending them to HR and bringing their own copy to the later workshop in their managerial group (described below). Furthermore, managers are responsible for ensuring actions and engagement in the implementation of specific action plans.

The next step is for the team managers to conduct their own *2.5-hour first-level managerial group workshops*. The purpose of both the first-level manager workshop and the later second- and top-level manager workshops are twofold:

1. To provide managers with an overview of identified positive and problematic work environment aspects and action plans formulated by employees during the Delphi dialogues to identify cross-cutting themes and, if necessary, create new action plans or escalate the identified problems they could not address themselves to a higher management level.
2. To employ the Delphi dialogue tool among themselves to identify additional positive or problematic aspects relevant for each manager and the management team. This process was to be facilitated by the team manager.

In accordance with the train-the-trainer principle [59], intervention activities are to be coordinated and facilitated by members of the organization to ensure that the intervention is organizationally anchored. The research group was available for both formal and informal inquiries to assist HR members during the intervention period.

#### Team dialogues – the Delphi method

A core component of the employee- and manager-level workshops is structured team dialogues based on the Delphi dialogue method [58]. A Delphi dialogue tool is used to identify negative and positive aspects of the working environment through the involvement of all workshop participants. The dialogue is initiated by asking each employee to fill out a form where he or she is asked to write down at least three positive aspects of his/her psychosocial work environment and three aspects that can be improved/changed. The form is then passed around to the other employees, who are instructed to put a cross next to the statements they agree with. The process facilitator (the team manager) guides the process of establishing a shared overview by building a list of positive and problematic aspects, starting with those statements that obtained the largest agreement. The overview is written on a large poster or blackboard. The final step involves selecting 2–3 aspects for further consideration. Employees are instructed to prioritize three topics on the posters with a cross. A cross means that the topic according to the employee should be prioritized here and now. The topics with the most votes are discussed by the group to reach a mutual understanding and consensus of their importance.

#### IGLO action plans

Following the dialogue process, the planned agenda for each workshop is to develop specific IGLO action plans. IGLO is presented to participants as an abbreviation for **I**ndividual, **G**roup, **L**eader and **O**rganization and as a model and method to understand both problems and solutions at different levels of an organization [26]. Employees are instructed to develop an IGLO action plan to address each prioritized topic. This means discussing which activities are needed at each of the four organizational levels to improve the specific topic of concern. IGLO action plans can also include a request for further discussion at higher organizational levels (escalation).

Apart from conducting their own risk assessment, the managerial group workshops at various levels similarly focus on developing action plans based on the issues already identified in the participatory employee workshops and focus on the potential initiatives that could most appropriately be decided at the managerial level in question. In addition, at the top-level managerial group workshop, the participants are offered a statistical overview of the action plans developed throughout the organization based on the HR members’ registrations, as mentioned above.

#### Materials used during training

Materials developed by the project research team to guide HR and managers in how to implement the specific intervention activities consisted of:

1. A shared slideshow presentation to be used at all employee and manager workshops.
2. The video presentation “What is the psychosocial work environment?” intended to provide knowledge about the main work environmental factors contributing to work stress as a basis for discussions of the participants’ work environment.
3. Supporting materials and guidelines: Presentation of the Delphi method, Delphi form, Delphi facilitator guide, IGLO action plans, and an overview of tasks and responsibilities.

An overview of the agendas for the different seminars and workshops is shown in Fig. 2.

### Program theory

**Fig. 3:**
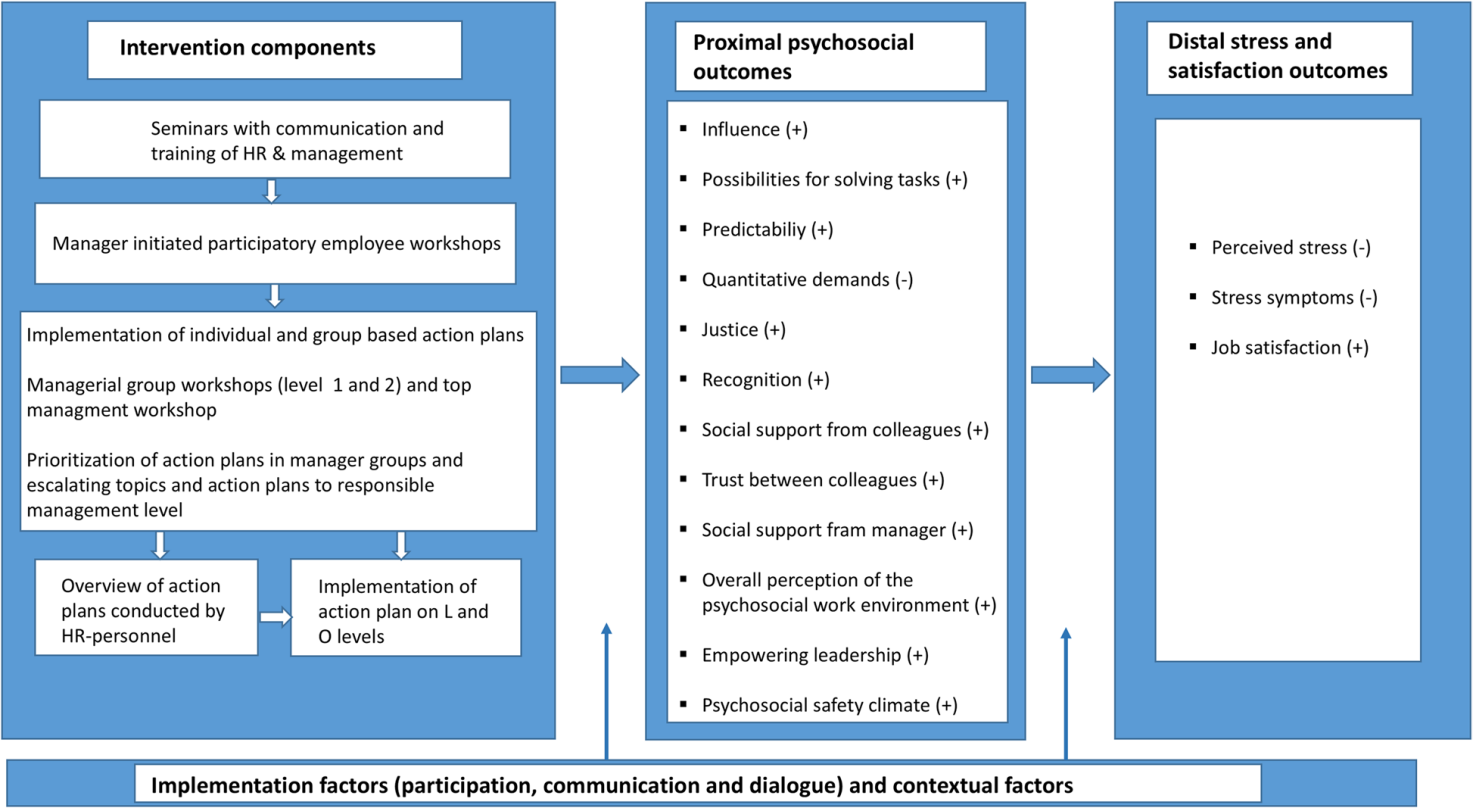
Program theory of expected mechanisms and changes

Figure 3 below outlines the program theory of the intervention to illustrate the expected order of change mechanisms and outcome changes [25]. The left side of the figure describes the different intervention activities as previously described. The right side of the figure illustrates the expected effect on outcomes related to the psychosocial work environment, stress and satisfaction. In general, the intervention was expected in the first instance to either increase psychosocial resources and/or reduce demands (proximal outcomes). Given the project’s focus on reducing perceived stress, particularly relevant demands and resources are the overall level of quantitative demands and factors associated with the demand-control-support model [16] (predictability, influence, possibilities for solving tasks, collegial and managerial social support, collegial trust), and factors associated with the effort-reward imbalance model [17] (justice, recognition). Such changes are expected to entail increased employee satisfaction with the psychosocial work environment. In addition, it was expected that the employees’ assessment of the PSC would improve since the intervention addressed all levels of the organization, the intervention was highly participatory, and the implementation of action plans was expected to lead to increases in empowering leadership. To the extent that the proximal outcomes were affected, we expected the intervention to lead to various distal outcomes, specifically reduced perceived stress and symptoms, and increased job satisfaction. While the main endpoints of the intervention were the stress outcomes, during the development of the program theory, it was decided to include job satisfaction as a supplementary distal outcome since many elements of the intervention aimed at promoting resources which could lead to enhanced satisfaction in line with the theoretical framework of the JD-R model. Symbols of + and – indicate an expected decrease or increase in the outcome measure.

### Data collection

Distal and proximal outcome measures as well as process evaluation measures were assessed by a questionnaire survey conducted before (May–June 2019) and after the implementation phase of the intervention (November–December 2020). All questionnaire data were collected with the Research Electronic Data Capture electronic data capture tools (Redcap) hosted at Aarhus University [60]. REDCap is a safe, web-based tool designed with the purpose of assisting data capture for research studies. The questionnaires were e-mailed to the participants through REDCap prior to the initiation of HR workshops and after the action plan implementation period (please see Fig. 2 above). Finally, all action plans were collected from HR to assess to which degree actions were actually implemented. The project also comprised a qualitative evaluation based on interviews that were conducted during and following the implementation phase. Results from the qualitative evaluation are reported elsewhere.

### Outcome measures – proximal outcomes 

Central aspects of the psychosocial work environment were measured by selected scales and single items from the Danish Psychosocial Questionnaire (DPQ). Where relevant, a scale was calculated, and scores on each aspect were recalculated from 0–100 as recommended [61]***.***

*Quantitative demands* were measured with four items, for example, “How often is it the case that you do not have time to complete all your work tasks?” and “How often do you receive unscheduled work tasks that place you under time pressure?.” All items were measured with a five-point Likert scale ranging from “Never/almost never” (1) to “Always” (5) Cronbach's alpha for baseline and follow-up was 0.86 and 0.90 .

*Predictability* at work was measured with the question “Do you receive timely information about important decisions, changes and plans for the future at your workplace?.”

*Influence at work* comprised four items, such as “Do you have any influence on how you carry out your work tasks?” and “Do you have sufficient authority to deal with the responsibilities you have in your work?.” Cronbach's alpha for baseline and follow-up was 0.85 and 0.89.

*Possibilities for performing work tasks* comprised four items: “Do your working conditions allow you to carry out your work satisfactorily?” and “Do you have the tools you need (e.g., technical assistive devices, tools, machinery, IT solutions, etc.) for you to do your job satisfactorily?” Cronbach's alpha for baseline and follow-up was 0.80 and 0.82.

*Social support from colleagues* was measured with four items such as “Can you get practical help with your work from colleagues if you need it?” and “Can you talk to your colleagues about it if you experience difficulties at work?.” Cronbach's alpha for baseline and follow-up was 0.82 and 0.83.

*Social support from management* was measured with four items such as “Can you get practical help with your work from your immediate supervisor if you need it?” and “Can you talk with your immediate supervisor about difficulties you experience at work?” All items were measured with a five-point Likert scale ranging from “Never/almost never” (1) to “Always” (5). Cronbach's alpha for baseline and follow-up was 0.90 and 0.91.

*Trust of colleagues* was measured with the item “Can you express your views and feelings to your closest colleagues?” 

*Justice* was measured with the item “Does the management at your workplace treat you fairly?.

*Recognition* was measured with the question “Are your efforts recognized and appreciated at your workplace?.”

All of the above were measured with a five-point Likert scale ranging from “to a very small extent” (1) to “to a very large extent” (5).

*Overall perception of the psychosocial work environment* was measured with a single item: “Overall, how satisfied are you with the psychosocial work environment at your workplace?.” Respondents were asked to answer on a scale from 0 to 10, where 0 denotes the lowest possible assessment.

*Psychosocial safety climate* was measured using the four-item version of the Psychosocial Safety Climate questionnaire [62]: ‘Senior management shows support for stress prevention through involvement and commitment’’; ‘‘In practice, the prevention of stress involves all levels of the organization’’; ‘‘In my organization my contributions to resolving occupational health and safety concerns regarding psychological wellbeing are listened to’’; and ‘‘Participation and consultation in occupational health and safety issues occurs with employees, unions and occupational health and safety representatives’’. Respondents were asked to answer on a 5-point Likert scale ranging from strongly disagree (1) to strongly agree (5), with respondents’ scores being summed on a scale (4-20) Cronbach's alpha for baseline and follow-up was 0.89 and 0.89.

*Empowering leadership* was measured with four items from the empowering leadership questionnaire with reference to the experience of whether the manager [63]: “encourages work group members to express ideas/suggestions”, “listens to my work group's ideas and suggestions”, “uses my work group's suggestions to make decisions that affect us” & “gives all work group members a chance to voice their opinions”. All items were measured with a five-point Likert scale ranging from “none” (1) to “very much” (5). Cronbach's alpha for baseline and follow-up was 0.94 and 0.94.

### Outcome measures – distal outcomes

*Perceived stress* was measured with the Danish consensus version of the 10-item Perceived Stress Scale (PSS10), which was developed to estimate the extent to which an individual finds his or her life to be unpredictable, uncontrollable and overwhelming [64]. Each item is scored on a 5-point Likert scale ranging from 0 (never) to 4 (very often), and the total score (range 0–40) was calculated as the sum of item scores after converting items 4, 5, 7 and 8, which were formulated as positive questions. Cronbach's alpha for baseline and follow-up was 0.91 and 0.89.

*Stress symptoms* were measured with a validated single item question where stress was defined as “feeling tense, restless, nervous, anxious or having difficulty sleeping at night because of thoughts about problems all the time” [65]. Participants were asked if they felt this kind of stress on these days. The item was answered on a 5-point Likert scale varying from 1 (not at all) to 5 (very much).

*Job satisfaction* was measured with a single item: “Overall, how satisfied are you with your job?” Respondents were asked to answer on a scale from 0 to 10, where 0 denotes the lowest possible assessment.

### Quantitative process evaluation

Data related to the process evaluation were collected through baseline and the follow-up questionnaires. Questions were self-formulated to address readiness for change, more specifically to what extent respondents 1) wanted to be involved more with regard to the psychosocial work environment, 2) if there was a need for a project that focused on the psychosocial work environment, and 3) if the person believed that the psychosocial work environment in the workplace could be improved. The answer categories were measured with a five-point Likert scale ranging from “to a very small extent” (1) to “to a very large extent” (5).

Questions at follow-up addressed whether respondents had participated in the workshop, how they experienced the workshops in terms of addressing relevant work environment aspects and the development of action plans, and their overall satisfaction with the workshops. Additional questions focused on whether action plans were implemented, whether they led to work environment improvements, and to what extent the implementation was prioritized by the manager, among other topics. The wording of all questions and response categories are featured in Table 4 below.

In addition to questions on implementation, participants were asked if changes related to the COVID-19 crisis had impacted the psychosocial work environment independently of the intervention since COVID-19 occurred during the intervention and follow-up period and to what extent any such changes had influenced the project.

As mentioned above, data on the implementation of the IGLO action plans were collected by HR. The HR department was to hand over the collected data excel sheet to the researchers by the end of the intervention.

### Statistical analyses

Baseline characteristics are presented by descriptive statistics. Distributions of outcome measures are presented by mean numbers, percentages and standard deviations. Comparisons of baseline characteristics, demographics and baseline outcome measures between dropouts and completers were conducted with chi-squared analyses and unpaired t tests.

We estimated change over time (baseline to follow-up) by employing a linear mixed model with a fixed effect of time and a random level for each individual, i.e., adjusting for the variation between individuals while using all data. The model estimates the mean difference between baseline and follow-up. Instead of leaving out data from respondents who dropped out, the model takes all data into account when estimating the mean difference between baseline and follow-up. Crude and adjusted models were performed: Model 1 adjusted for workplace within the municipality. Model 2 adjusted for workplace and age, and Model 3 adjusted for workplace, age and gender. Similar to other studies we adjusted for workplace, age and gender [66–68]. This was decided prior to conducting analyses.

In addition, a sensitivity analysis was conducted including only respondents who had responded at both baseline and follow-up using a paired t test. In exploratory analyses, linear regression analyses adjusting only for baseline of the outcome were conducted to examine the difference in pre-post changes between those who participated in workshops and those who did not.

Effect sizes based on the fully adjusted model were calculated as Cohen’s d as the mean change from baseline to follow-up divided by the square root of the total dispersion from the mixed model.

All statistical analyses were performed using the statistical software package Stata, version 17 (Stata Corporation, College Station, Texas, USA).

## Results

### Participants

In total, ten workplaces/departments (*N* = 542) within the municipality were included to participate; out of these, three units dropped out during the study: one daycare center, one school and one administrative unit. Reasons for dropping out were reported as busyness, change of manager, and conflict between the workplace and HR, which, as noted, was central to the completion of the intervention. Across the participating workplaces/departments, 322 respondents were invited to answer the baseline questionnaire in 2019, and 235 (73%) answered. At follow-up in 2020, 268 respondents were invited to participate, and 130 (49%) answered the questionnaire.

Table 1 illustrates the characteristics of the 235 participants who agreed to participate at baseline. Those who participated at follow-up are also included in the table. In total, 139 respondents participated only at baseline, 34 respondents participated only at follow-up, and 96 respondents participated at both baseline and follow-up. The majority of respondents were women, married and had medium higher education. The largest occupational groups were teachers and daycare workers, and the majority of the respondents were employed in permanent positions and had worked at their current workplace between 1–5 years. At baseline, 11% reported that they had been on sick leave for more than 2 weeks during the previous year.

### Completers versus dropouts

There were significantly more men among the dropouts than among the completers (*p* = 0.025). There was no significant difference between completers and dropouts with regard to age, civil status, education, job position, years in current workplace or the degree of temporary or permanent employment or baseline outcome scores, except for quantitative demands where dropouts reported significantly lower demands compared to completers (39.89 versus 46.63) (*p* = 0.02).

### Mixed models

Table 2 below shows the results from the mixed models using all observations in analyzing the various outcomes. Although some respondents had not participated in the workshops themselves, we chose to include their responses since the workshops and any implementation of IGLO action plans might have affected all employees at the particular workplace unit. In the crude model, a significant decrease in quantitative demands was observed from baseline to follow-up, as well as small significant improvements on possibilities for solving work tasks, social support from closest manager, perceived psychosocial work environment, psychosocial safety climate and empowering leadership. Among the distal outcomes, an improvement was observed in job satisfaction.

**Table 2:** Change from before to after the intervention on outcomes related to the psychosocial work environment, satisfaction and stress

Measure (range)	Mean change from baseline to follow-up	95% CI	p-value	Mean change adjusted for workplace	95% CI	p-value	Mean change adjusted for workplace and age	95% CI	p-value	Mean change adjusted for workplace, age and gender	95% CI	p-value	Cohen's d
Influence at work (0 -100)	0.93	-1.57 to 3.43	0.47	1.10	-1.42 to 3.63	0.39	0.72	-1.82 to 3.26	0.58	0.67	-1.87 to 3.20	0.61	0.04
Possibilities for solving work tasks (0-100)	2.81	0.30 to 5.32	0.03	2.92	0.39 to 5.44	0.02	2.5	0.04 to 5.05	0.05	2.46	-0.09 to 5.01	0.06	0.15
Predictability at work (0-100)	3.12	-0.49 to 6.74	0.09	3.47	-0.21 to 7.16	0.06	3.61	-0.10 to 7.33	0.06	3.44	-0.29 to 7.16	0.07	0.18
Quantitative demands (0-100)	-5.75	-9.65 to -1.85	<0.01	-6.31	-10.25 to -2.36	<0.01	-5.84	-9.85 to -1.83	<0.01	-5.84	-9.85 to -1.82	<0.01	-0.27
Justice (0-100)	1.98	-1.27 to 5.23	0.23	2.49	-0.80 to 5.79	0.14	2.54	-0.79 to 5.88	0.13	2.59	-0.74 to 5.93	0.13	0.13
Recognition (0-100)	-0.01	-0.15 to 0.14	0.95	-0.03	-0.18 to 0.13	0.74	-0.003	-0.16 to 0.15	0.97	-0.01	-0.16 to 0.14	0.91	-0.01
Social support from colleagues (0-100)	0.60	-1.75 to 2.96	0.62	0.71	-1.67 to 3.10	0.56	0.84	-1.58 to 3.26	0.5	0.77	-1.66 to 3.19	0.54	0.06
Trust of colleagues (0-100)	-0.12	-3.58 to 3.34	0.95	0.03	-3.49 to 3.56	0.99	-0.07	-3.67 to 3.53	0.97	-0.25	-3.84 to 3.35	0.89	-0.01
Social support from closest manager (0-100)	3.89	0.22 to 7.57	0.04	3.73	-0.01 to 7.48	0.05	3.55	-0.25 to 7.34	0.07	3.58	-0.21 to 7.36	0.06	0.18
The perceived psychosocial work environment (0-10)	0.55	0.22 to 0.88	<0.01	0.58	0.25 to 0.91	<0.01	0.56	0.22 to 0.89	<0.01	0.55	0.22 to 0.89	<0.01	0.28
Empowering leadership (4-20)	1.09	0.43 to 1.76	<0.01	1.10	0.42 to 1.78	<0.01	1.09	0.41 to 1.78	<0.01	1.11	0.42 to 1.80	<0.01	0.32
Psychosocial safety climate (4-20)*	-0.62	-1.14 to -0.09	0.02	-0.60	-1.13 to -0.07	0.03	-0.57	-1.10 to -0.04	0.04	-0.55	-1.08 to -0.02	0.04	-0.18
Perceived stress (0-40)	0.02	-1.19 to 1.22	0.98	-0.00	-1.22 to 1.21	1.00	0.21	-1.02 to 1.44	0.74	0.19	-1.03 to 1.41	0.76	0.03
Stress symptoms (0-100)	1.52	-3.06 to 6.10	0.52	1.23	-3.38 to 5.85	0.60	2	-2.69 to 6.69	0.4	1.8	-2.86 to 6.47	0.45	0.07
Job satisfaction (0-10)	0.31	0.04 to 0.58	0.02	0.36	0.09 to 0.64	0.01	0.33	0.05 to 0.60	0.02	0.32	0.04 to 0.6	0.03	0.21

*A low score indicates a higher psychosocial safety climate

In the fully (workplace, age, and gender) adjusted model, the improvements in quantitative demands, the perceived psychosocial work environment, the psychosocial safety climate and empowering leadership remained significant, while borderline significant improvements were observed for possibilities for solving work tasks, predictability at work, and social support from closest manager. No improvements were found with regard to the distal stress outcomes but a small improvement in job satisfaction remained. The overall largest effect sizes in the full model were found for quantitative demands, empowering leadership, the perceived psychosocial work environment and job satisfaction. All effect sizes can be categorized as small in terms of Cohen’s d.

**Table 3:**
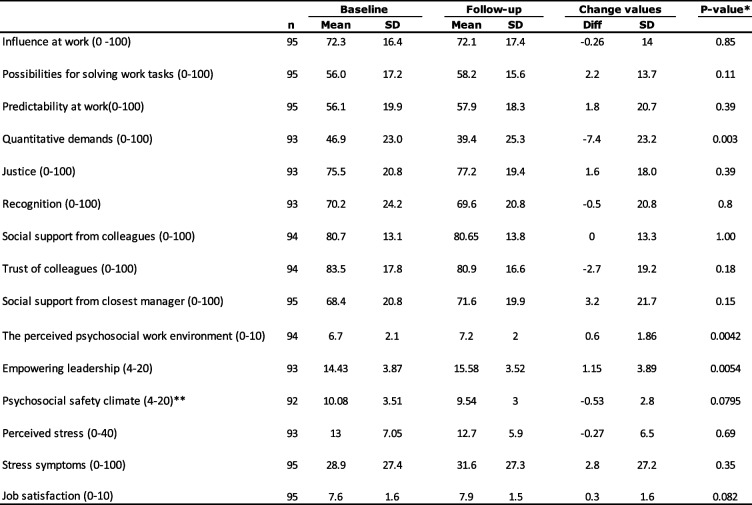
Changes in outcomes from baseline to follow-up among participants that responded to both baseline and follow-up measurements

*Paired ttest

**A low score indicates a higher psychosocial safety climate

Table 3 shows the results of the sensitivity analyses based on paired t-tests conducted on respondents who participated at both baseline and follow-up. These results show similar trends to the fully adjusted mixed model with regard to no improvement in perceived stress or stress symptoms, but positive trends on quantitative demands, the perceived psychosocial work environment, empowering leadership and job satisfaction. 

### Process evaluation and action plans

#### Baseline evaluation of readiness for change

Of those who responded to the baseline questionnaire (*N* = 235), 79 respondents (33.6%) were interested in becoming involved with regard to the psychosocial work environment to a high or very high extent. Ninety-three (39.6%) answered to some degree, while 59 respondents (25.1%) answered to a less or very low degree. Four individuals did not answer the question (1.7%).

Asked if they believed that there was a need for a project focusing on the psychosocial work environment 68 respondents (28.9%) answered to a high or very high degree, 99 (42.1%) answered to some degree, and 63 (26.8%) answered to a low or very low degree. Five individuals did not answer the question (2.1%).

Finally, participants were asked to what extent they believe that it is possible to improve the psychosocial work environment at their workplace. Eighty (34.04%) respondents answered to a very high or high degree, while 111 (47.2%) believed that change was possible to a certain extent. Thirty-nine (16,6%) answered to a small or very small degree. Five individuals did not answer the question (2.1%).

#### The delivered intervention

**Fig. 4:**
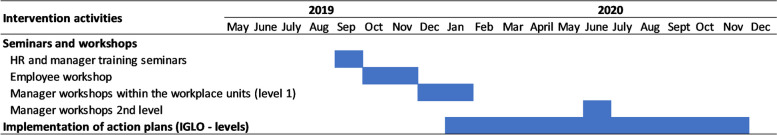
Timeline over intervention activities

Figure 4 illustrates the intervention activities as they actually took place during the intervention in the municipality. The entire intervention process lasted a year and three months, as illustrated in Fig. 4. Some aspects were not conducted entirely as planned. While manager levels 1 and 2 completed their workshop, the top management of the organization did not conduct their work-shop as planned, and it is therefore not shown below.

The development and implementation of action plans on all IGLO levels was an essential part of the intervention, and therefore, all action plans were to be collected by HR. Categorizations of the developed action plans are shown in Supplementary (File 1). The data on whether action plans were followed up were incomplete, as the HR department did not facilitate all parts of the intervention setup as planned. However based on the collected data, 37 action plans were developed in total. The main themes in the action plans (apart from “other things”) were “cooperation” and “colleague relations—professional aspects” along with “high demands—workload or pace,” indicating that these themes were essential for improving the psychosocial work environment in some workplace units. Additionally, “work organization and predictability” and “management” were each addressed in four action plans. Negative events such as bullying and workplace violence were apparently not addressed in the action plans.

In total, the action plans contained 127 initiatives for different IGLO-levels. The Individual, group and leader levels were addressed with 37 initiatives each while the organizational level was addressed in 16 initiatives.

#### Follow-up evaluation of implementation and engagement

Table 4 below provides an overview of the perception of the intervention and the degree of implementation among those who responded to the follow-up questionnaire.

**Table 4:**
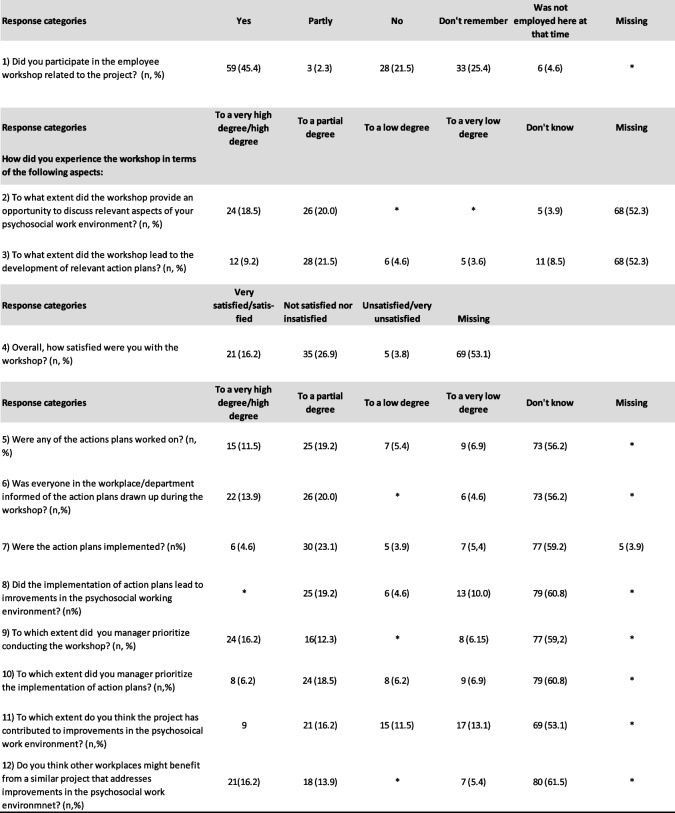
Process questions on workshop completion, implementation of action plans and communication among those who responded to the follow-up questionnaire (*n* = 130)

*Cells with less than 5 observations

Among those respondents who participated in a workshop, most respondents stated a neutral or positive degree of satisfaction and that the workshops to a high degree or partially comprised discussions of relevant working environment aspects and led to the development of action plans.

Since workshop nonparticipants could in principle still participate in activities related to the action plans after the workshop, these respondents were still presented with the items related to the implementation process. However, the large proportion of “don’t know” responses suggests that only a smaller group of employees had participated in the implementation or received substantial information on its progress. Among those who did provide a response assessing the degree of implementation, the distribution of responses suggests that action plans were generally only partially implemented, with a moderate to high degree of effort, supported by a moderate degree of prioritization by managers. The respondents generally assessed the action plans to have had a moderate to low impact on the psychosocial work environment. Looking at only those who reported that they had fully or partly participated in the workshops, 47% responded that they believed other workplaces may benefit from the implementation of a similar intervention.

To examine whether participation in the workshops was associated with pre-post changes, a number of exploratory sub-analyses were conducted on respondents that answered both at baseline and follow-up. In linear regression analyses adjusting only for baseline of the outcome, we examined if there was a difference in pre-post changes between those who participated in workshops and those who did not. We found only a very small and nonsignificant between-group difference in pre-post changes with regards to perceived stress (difference = −0.15, 95% CI: −2.33 – 2.03, *p* = 0.893) as well as in all other measures (not shown). The largest difference was observed in social support from closest manager, where those who did not participate showed a slightly larger pre-post improvement (difference = 3.81 95% CI: −3.57—11.18, *p* = 0.308) compared to those who took part in the workshops.

#### The role of COVID-19 on the psychosocial work environment

In addition to the implementation questions above, we also asked participants if changes related to COVID-19 had had an impact on the psychosocial work environment independent of the intervention. Among those who participated in follow-up, 9 (6.9%) respondents answered that they had experienced improvements in the working environment, 31 (23.9%) respondents had experienced worsening, and 39 (30%) respondents had experienced both improvements and negative changes. Thirteen (10%) respondents had not experienced any changes, and 39 (30%) did not respond. We also asked participants at follow-up to which extent workplace changes implemented in relation to the corona crisis had influenced the project. Thirty-six (27.7%) answered that such changes had affected the project to a high or very high degree, 38 (29.2%) respondents answered to a low degree, and 13 (10%) answered to a very low degree. Forty-three (33%) did not respond.

## Discussion

The primary purpose of this study was to evaluate the effectiveness of an organizationally anchored multilevel intervention on pre-post changes with regard to the psychosocial work environment and stress outcomes. The study tested the intervention in a municipality setting comprising mainly schools and daycare settings. At follow-up, we found small improvements in some of the proximal outcomes, specifically in workplace resources such as the overall perception of the psychosocial work environment, the psychosocial safety climate, and empowering leadership. Possitive trends were also observed for possibilities for solving work tasks, predictability at work, and support from closest manager although these trends were borderline significant when adjusting for workplace, age, and gender. We also observed a small decrease in quantitative demands from baseline to follow-up. We found no improvements in perceived stress or stress symptoms but a positive tendency with regard to job satisfation. Moreover, we did not find substantial or statistically significant changes in influence at work, justice, recognition, social support from colleagues, or trust in colleagues in either model. Overall, we observed tendencies corresponding with some of our theoretical expectations about proximal outcomes (see Fig. 3), although effect sizes were small. However, for other hypothesized relationships for example with regard to stress, we did not find empirical support. The results generally suggest only small effects on the expected outcomes. We may attribute the scarce confirmation of the total program theory to a number of methodological and intervention-related limitations, which we will discuss further below.

In support of the intervention, the data revealed that employees and their managers developed numerous action plans and more than 120 initiatives to improve the work environment as a part of the intervention. Moreover, the workshop participants that responded to the follow-up measurement generally reported neutral to positive assessments of the workshops and reported having worked on the implementation of action plans from a very high to a partial degree. We know from a similar study in the teaching sector that there can be large variations in the implementation of participatory organizational level occupational health interventions [69]. In this light, it makes sense to enquire further into the pattern of results. In all models, a decrease in quantitative demands was found. This may be a result of solved workplace problems that operated as obstacles to meeting quantitative demands. In addition, several of the action plans pertained to solving problems with quantitative job demands (see Supplementary: File 1). There was a borderline significant increase in predictability after the intervention, which may be interpreted as a result of the intervention to the extent that the process increased sharing of information and enhanced clarity about work among employees and managers. The results in the full model (Table 2) and in the sensitivity analysis including only those that answered at both timepoints (Table 3) showed improvements in the psychosocial safety climate, the perceived psychosocial work environment and empowering leadership. These dimensions may be understood as a response to experiencing employee participation and focus on wellbeing from one’s immediate manager as a part of the intervention. Overall, a positive interpretation of the results may suggest that the intervention was a good opportunity to solve problems and share information and potential solutions, which may have improved overall perception of the psychosocial work environment and led to fewer quantitative demands and an increase in job satisfaction. This is somewhat in line with a Cochrane review of four organizational interventions for reducing stress among teachers that found low-quality evidence of improvements in teachers’ well-being and retention rates [39].

However, the results also fail to confirm changes after the intervention on other hypothesized key parameters. We could not provide results to suggest that the intervention facilitated trust and social support among colleagues. This is surprising when considering that 10 out of 37 action plans were related to either social or professional aspects of cooperation. These results are unexpected because solving problems together would have the potential to strengthen coworker ties. However, this may be a result of social support being high in the organization even prior to the intervention. In addition, the analyses could not confirm that the intervention improved recognition, justice, or lowered stress (perceived and experiencing symptoms). More unexpectedly, the intervention did not seem to enhance influence at work, although this is a key dimension of the workshops. However, the lack of results of our participatory approach on some of our outcomes (although participative approaches have been widely called for) seem in accordance with a recent review [70] of organizational level interventions in healthcare. This review found that participatory interventions were less predictable, and that this could be related to the main focus being placed on the participatory process (workshops, meetings), while it is rather unclear if the issues that are identified and potential solutions are actually implemented [70]. In addition, only one study out of five participatory intervention studies measuring stress found an overall effect on stress, while a second study found a delayed effect on stress after 12 and 18 months. Thus, we cannot rule out that the implementation of action plans and their potential effect on stress outcomes may take time to unfold beyond the follow-up times of the current study.

The exploratory analyses of the subsample that answered both baseline and follow-up, did not show any substantial differences in pre-post changes between those that had participated in workshops and those that had not indicating that taking part in the workshop in itself did not enhance improvements. However, these analyses should be interpreted carefully since close colleagues not taking part in the workshop could still be impacted by the workshop and the implementation of action plans potentially making it difficult to detect a difference. The largest difference was observed in the outcome of social support from the closest manager, with those who did not participate in workshops experiencing a larger improvement in manager support. A cautious interpretation of this could be that participation and the following work on action plans may have been demanding for participants, potentially leading them to provide less favorable assessments of manager support. It’s also possible that the workshops increased participants' awareness and expectations of manager support and the role of the manager in implementing changes, which were then disappointed leading to smaller improvements in those directly involved in workshops than those who were not. However, other factors, such as local changes within the organizations, could also have influenced these results.

Estimates for possibilities for solving work tasks and social support from closest manager became borderline significant when adjusting for workplace, age, and gender. Some may argue that this is too strict an adjustment, however covariates where chosen prior to conducting the analyses based on the existing literature. We adjusted for workplace since climate and culture at individual workplaces may influence how employees cope with stress [71] which may cause a clustering effect that should be accounted for. Age and gender have been associated with work-related stress in several studies [5, 72, 73] although mixed results have been found with regard to gender [73]. In the current study adjusting for gender hardly altered the results.

The mixed results and the implementation challenges of the intervention resemble the overall pattern found for effects of organizational interventions [39, 40]. A recent systematic overview of reviews found moderate-quality evidence that organizational interventions focused on improving aspects of the psychosocial work environment could lead to positive effects in the psychosocial work environment or employee wellbeing. Positive results were seen in studies that facilitated workgroup activities enhancing communication and support as well as in studies using a participative approach to enhance process aspects in the work environment and core tasks. The same systematic overview found inconclusive evidence of whether organizational-level interventions could also reduce stress [28]. These results seem consistent with the results of our study, supporting the assumption that participatory organizational intervention may yield positive effects on proximal work environment outcomes to a greater extent than more distal health outcomes such as perceived stress. However, the current study differs from many other organizational intervention studies by the design to target all levels of the organization as well as by incorporating the possibility of escalating work environmental problems from one management level upward to the manager level with the appropriate decision authority. While multilevel interventions have been extensively called for in the literature [22, 26, 74], actual attempts to conduct a multilevel organizational workplace intervention spanning several leadership levels remain scarce in the literature. This may be a result of the high level of complexity involved when targeting several organizational levels at the same time. For example, some highly demanding aspects of this intervention are facilitating processes where employees and managers work together to solve problems in the psychosocial work environment and implement solutions to those problems [28], and the demanding tasks of HR to monitor the specific types and the escalation and implementation of action plans.

### Intervention-related limitations

A number of limitations to the study can be found in the intervention model and its implementation. The analysis of the action plans reveals that the intervention was applied with many different themes corresponding to a diversity of our parameters. Seven out of the 37 reported action plans pertained to issues outside of the scope of the present project. Since the workplaces seem to have worked with very different issues, it is unlikely that all the workplaces on average will experience improvement in the same parameters. The potential mismatch in participatory interventions between the outcome measures (chosen a priori by the researchers) and the action plans developed in the intervention by the employees has also been mentioned by others [75, 76]. Moreover, 35 respondents of the quantitative process evaluation rated that action plans were only partially or to a low degree implemented. This is in line with the findings from our qualitative process evaluation paper of this project (currently *under review*), which revealed multiple implementation challenges and that a key element of the intervention-the top-level manager workshop- was not conducted. Adding to the impression of a less than ideal implementation of the intervention, many respondents had not or did not remember if they participated in the workshop. This may be explained by there being approximately one year between the workshops and the follow-up questionnaire and by the nature of the type of work in the participating workplaces preventing all employees from participating at the same time (for example, in a daycare setting) and by employee turnover during the follow-up period. The intervention was quite demanding for the managers that were responsible for conducting the team dialogues and for implementing action plans at the team level. Even though the managers had been trained in the intervention tools, the implementation of action plans may have been too challenging for many managers as revealed by the process questions and considering the many daily tasks and high span of control that municipality managers often face. However, several studies and reviews have underlined the importance of line manager engagement when conducting and implementing organizational interventions [31, 77].

### Methodological limitations

A major contextual event occurred simultaneously with the implementation period of the intervention in the form of the global COVID-19 crisis. The crisis caused changes in the daily work lives of almost all public workers. Hence, changes in the perceived psychosocial working environment may have been caused by changes in work caused by the COVID-19 crisis itself and the way workplaces and society handled the crisis. At a general methodological level, the applied methods do not allow us to rule out the possibility of contextual contamination of the results. If the research design included a control group that was influenced by the same contextual influences, possible contextual confounding could have been excluded from the results. Control groups may, however, be difficult to establish as sufficiently comparable workplaces can be particularly difficult to encounter when conducting a multilevel intervention, where randomization of units at different organizational levels is not an option since all units are encompassed by the same top management [78].

We found no improvements in stress outcomes, but we observed positive changes in some proximal outcomes that are often related to work stress (e.g., quantitative demands). Here, we can argue that there may have been a floor effect since respondents had a relatively low level of perceived stress and stress symptoms at baseline. Hence, the selection of participants for stress preventive intervention studies may benefit from including workplaces with a need for stress reduction. Additionally, work-related mental health problems are likely to be caused by long-term imbalance between demands and resources, and these more distal outcomes may also take a longer time to improve after the intervention than proximal outcomes, meaning that a potentially longer follow-up period could be relevant. Thus, another potential limitation of the study lies in the relatively short follow-up period. Certain aspects of the intervention may require extended periods to fully implement – particularly those escalated to higher organizational levels, which may take significantly longer to address compared to issues that can be resolved immediately within a work team. As a result, there may be a time lag in the intervention’s ultimate effects, which our measurements may not have fully captured.

In general, the results should be interpreted with caution since response rates were lower than expected, especially due to attrition from baseline to follow-up. The main strength of the current study lies in developing, documenting, and testing a new type of theoretically supported multilevel intervention and evaluating its implementation and potential effectiveness. Conducting this intervention in other types of organizations where implementation to a higher extent can be ensured may improve the results.

## Conclusion

Overall, our study suggests that this multilevel organizational intervention was associated with small positive changes in the working environment. However, the study also highlights how a number of practical and methodological challenges exist in relation to conducting this type of multilevel intervention, and this compromised our ability to ensure that the intervention was carried out as intended and that the findings of the evaluation could be unambiguously attributed to the intervention itself. As a consequence, future studies seeking to assess the effectiveness of similar interventions should carefully consider design and evaluation approaches.

## Supplementary Information

Supplementary Material 1.

## Data Availability

The datasets used and/or analyzed during the current study are available from the corresponding author on reasonable request.

## Abbreviations

HR: Human Resource Department
IGLO: Individual, Group, Leader, Organization
PSC: Psychosocial Safety Climate
PSS10: The Perceived Stress Scale with 10 items
Redcap: Research Electronic Data Capture electronic data capture tools
