# Supplementary figures and images for: Can an organizationally anchored, multilevel intervention improve perceived stress and psychosocial factors in the workplace? A pre-post study assessing effectiveness and implementation
Source: BMC Public Health. 2025 Jan 30;25:384. doi: 10.1186/s12889-024-20801-5 (PMC11783957; doi:10.1186/s12889-024-20801-5)

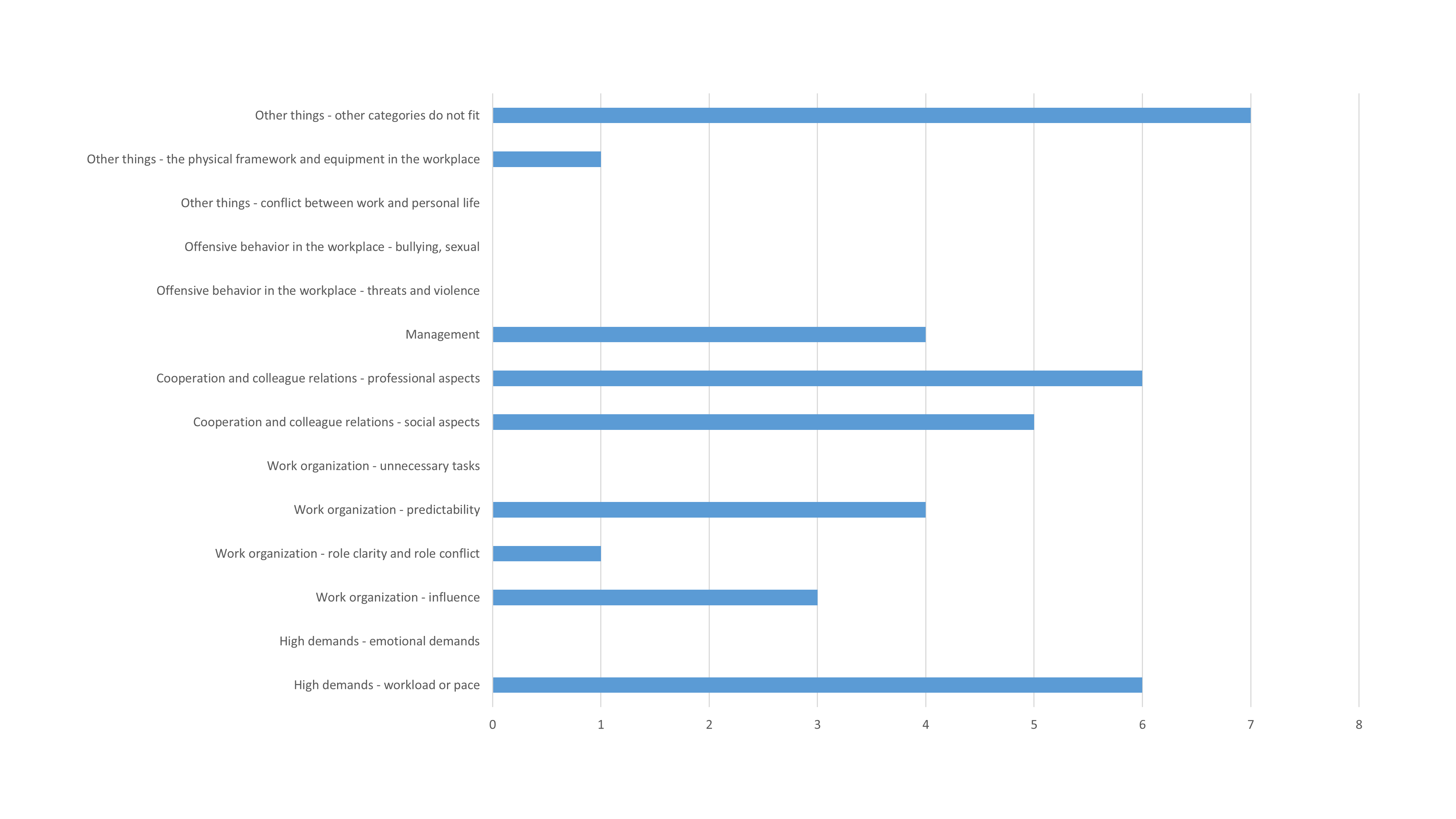

Supplement: Supplementary file 1 — Supplementary Material 1. [file 12889_2024_20801_MOESM1_ESM.tif]
